# Dynamics of Fusarium Mycotoxins and Lytic Enzymes during Pea Plants’ Infection

**DOI:** 10.3390/ijms22189888

**Published:** 2021-09-13

**Authors:** Lakshmipriya Perincherry, Monika Urbaniak, Izabela Pawłowicz, Karolina Kotowska, Agnieszka Waśkiewicz, Łukasz Stępień

**Affiliations:** 1 Department of Plant-Pathogen Interaction, Institute of Plant Genetics, Polish Academy of Sciences, 60-479 Poznań, Poland; murb@igr.poznan.pl (M.U.); kar.kotowska@gmail.com (K.K.); 2 Department of Plant Physiology, Institute of Plant Genetics, Polish Academy of Sciences, 60-479 Poznań, Poland; ipaw@igr.poznan.pl; 3 Department of Chemistry, Poznań University of Life Sciences, 60-625 Poznań, Poland; agnieszka.waskiewicz@up.poznan.pl

**Keywords:** CWDEs, mycotoxins, *Fusarium*, lytic enzyme gene expression, plant–pathogen interaction

## Abstract

*Fusarium* species are common plant pathogens that cause several important diseases. They produce a wide range of secondary metabolites, among which mycotoxins and extracellular cell wall-degrading enzymes (CWDEs) contribute to weakening and invading the host plant successfully. Two species of *Fusarium* isolated from peas were monitored for their expression profile of three cell wall-degrading enzyme coding genes upon culturing with extracts from resistant (Sokolik) and susceptible (Santana) pea cultivars. The extracts from Santana induced a sudden increase in the gene expression, whereas Sokolik elicited a reduced expression. The coherent observation was that the biochemical profile of the host plant plays a major role in regulating the fungal gene expression. In order to uncover the fungal characteristics in planta, both pea cultivars were infected with two strains each of *F. proliferatum* and *F. oxysporum* on the 30th day of growth. The enzyme activity assays from both roots and rhizosphere indicated that more enzymes were used for degrading the cell wall of the resistant host compared to the susceptible host. The most commonly produced enzymes were cellulase, β-glucosidase, xylanase, pectinase and lipase, where the pathogen selectively degraded the components of both the primary and secondary cell walls. The levels of beauvericin accumulated in the infected roots of both cultivars were also monitored. There was a difference between the levels of beauvericin accumulated in both the cultivars, where the susceptible cultivar had more beauvericin than the resistant one, showing that the plants susceptible to the pathogen were also susceptible to the toxin accumulation.

## 1. Introduction

*Fusarium* wilt is one of the most devastating diseases worldwide. Susceptible plant species range to several hundred, including economically important food crops such as tomatoes, sweet potatoes, legumes, melons and bananas [1]. Species such as *F. oxysporum* f. sp. *pisi*, *F. oxysporum* var. *redolens*, *F. poae, F. solani* and *F. avenaceum* are considered minor pathogens of peas [2,3]. Being a soil-inhabiting fungus, *Fusarium* can survive for more than 10 years, as its spores are thick-walled and very hard. Upon encountering a susceptible plant, it penetrates the roots and grows inside the vascular system, causing wilt by interfering with water movement [4]. The pea plants infected with *F. oxysporum* have a characteristic reddish orange color on the vascular and lower parts of the stem, with rare damage to the cortex. The symptoms also include yellow, brittle and rolled-up leaves, which is the characteristic feature of wilt [2]. However, *F. proliferatum* is capable of surviving without visible disease symptoms in the seed materials, contaminating it with fumonisins (FBs) [5]—a family of polyketide derivatives that are structurally similar to sphinganine compounds. FBs disrupt sphingolipid metabolism, causing different toxicological effects in humans, animals and plants [6,7]. It was found that pea extracts could reduce the biosynthesis of group B fumonisins and could limit the fungal biomass in *F. proliferatum* [8]. Our previous report showed that the extracts from the resistant pea variety Sokolik accumulate less/no fumonisin B_1_ and B_2_ in *F. proliferatum* strain PEA1 when compared to the susceptible variety Santana [9]. Additionally, the Sokolik extract could also considerably reduce the biomass of both *F. proliferatum* and *F. oxysporum.*

Another major characteristic of the *Fusarium* species is the production of various cell wall-degrading enzymes (CWDEs). The majority of the CWDEs are glycosyl hydrolases responsible for the degradation of plant cell wall polysaccharides [10]. These enzymes are encoded in the pathogen genomes as multigene families [11]. The lytic enzymes produced by plant–pathogenic fungi enable them to penetrate the plant cell wall and infect the host tissue. The key CWDEs produced are cellulases, pectinases, proteases and lipases, which contribute to the degradation of waxy cuticle and cell walls. With the help of those enzymes, the pathogen depolymerizes almost all components of plant cell walls, including cellulose, xylan, pectin, polygalacturonic acids and membrane proteins [12,13,14,15,16,17]. It was evident from our recent studies that both *F. proliferatum* and *F. oxysporum* have an increased activity of β-glucosidase, pectate lyase and xylanase when supplemented with the extracts of Santana and Sokolik. In order to understand the increased disease resistance mechanism of Sokolik, plant infection studies are necessary. It not only helps us to understand the plant part but also allows us to shed light on the different strategies adapted by the pathogens to establish themselves inside the host.

## 2. Results

### 2.1. Fungal Gene Expression Studies

The plant cell walls differ in detailed chemical composition and structural organization. However, their basic architecture constitutes cellulose microfibers of immense tensile strength inserted in a water-saturated matrix of various structural glycoproteins and polysaccharides, which explains why the fungal pathogen synthesizes a wide range of plant cell wall-degrading enzymes during its interaction with the host.

**Figure 1:**
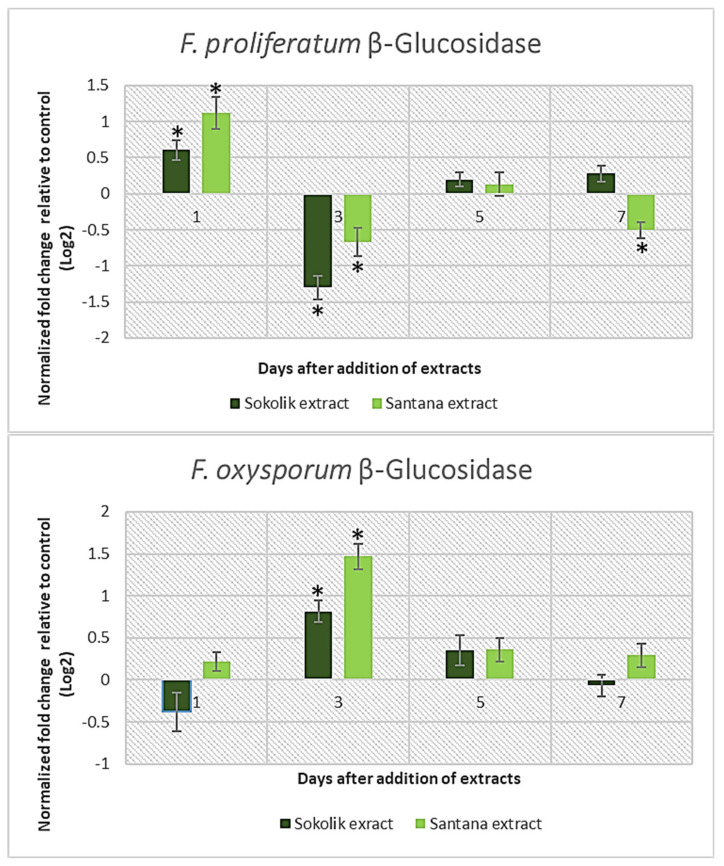
β−Glucosidase gene expression of *F. proliferatum* (PEA1) and *F. oxysporum* (1757 OX) from day 1 to day 7 after the addition of the Sokolik and Santana extracts. Error bars represent the standard error. * Statistically significant (*p <* 0.05).

β-glucosidases are considered the rate-limiting enzyme because of their responsibility to carry out the first step of lignocellulose hydrolysis, where cellobiose and short cellodextrins are converted into glucose. Therefore, β-glucosidase is essential for the complete hydrolysis of cellulose into glucose; it is also considered as an important component of the cellulose enzyme complex [18]. In our study, the expression of the β-Glucosidase-encoding gene was significantly enhanced in *F. proliferatum* PEA1 cultures immediately after the addition of both Sokolik and Santana extracts (Figure 1). Although being the susceptible variety, Santana could elicit an increased gene expression compared to Sokolik, irrespective of the pathogen species. The expression of *bgl1* was found to be reduced on the following days.

**Figure 2:**
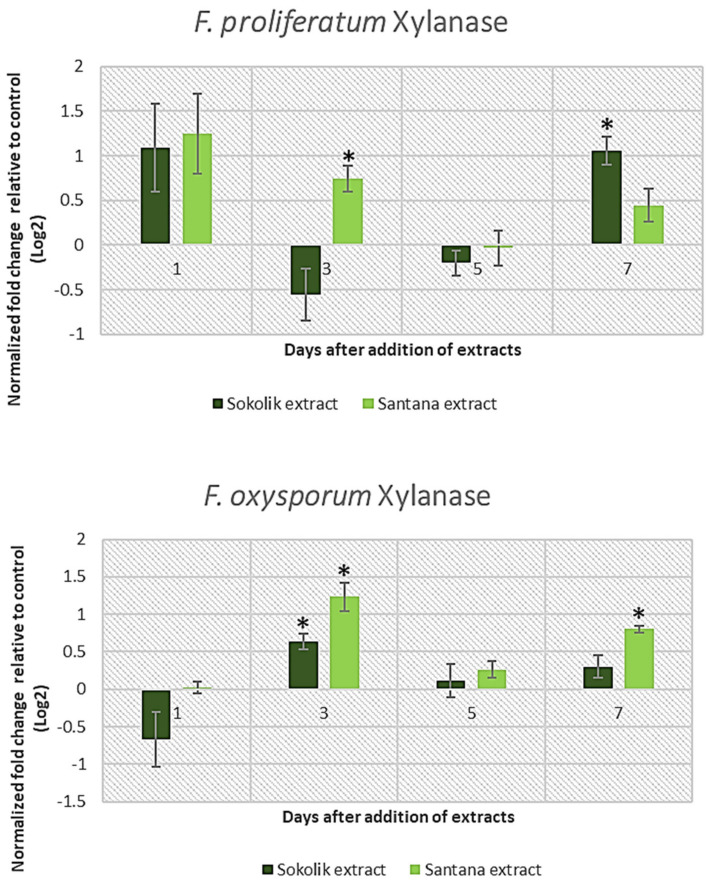
Xylanase gene expression of *F. proliferatum* (PEA1) and *F. oxysporum* (1757 OX) from day 1 to day 7 after the addition of the Sokolik and Santana extracts. Error bars represent the standard error. * Statistically significant (*p <* 0.05).

Hemicelluloses and pectins compose the two major classes of plant cell wall matrix polysaccharides synthesized in the Golgi apparatus. They are transported and secreted to the cell surface in small vesicles. Hemicelluloses are heteropolymers with side chains and are composed of pentoses (xylans), with units of mannose and glucose (mannans or glucomannans) or galactose (galactans) present alternatively [19]. Pathogens selectively degrade the hemicellulose with the help of xylanases, mannanases, arabinases and galactanases [20]. The expression of the xylanase gene in Santana extract-added cultures of PEA1 increased for days 1 and 3 and later reduced gradually (Figure 2). The cultures with the Sokolik extract showed increased xylanase gene expression on the last day of cultivation, similar to the pectate lyase gene. The characteristic sudden increase in the gene transcripts were not observed in the case of *F. oxysporum* strain 1757OX. Rather, the xylanase gene expression was found to be elevated from day 3 after the supplementation with extracts. A highly similar pattern of all the lytic genes’ expression was noticed in 1757OX for the entire culture period, indicating that all the pathways regulating the cell wall-degrading enzymes in *F. oxysporum* are activated with a similar pattern.

**Figure 3:**
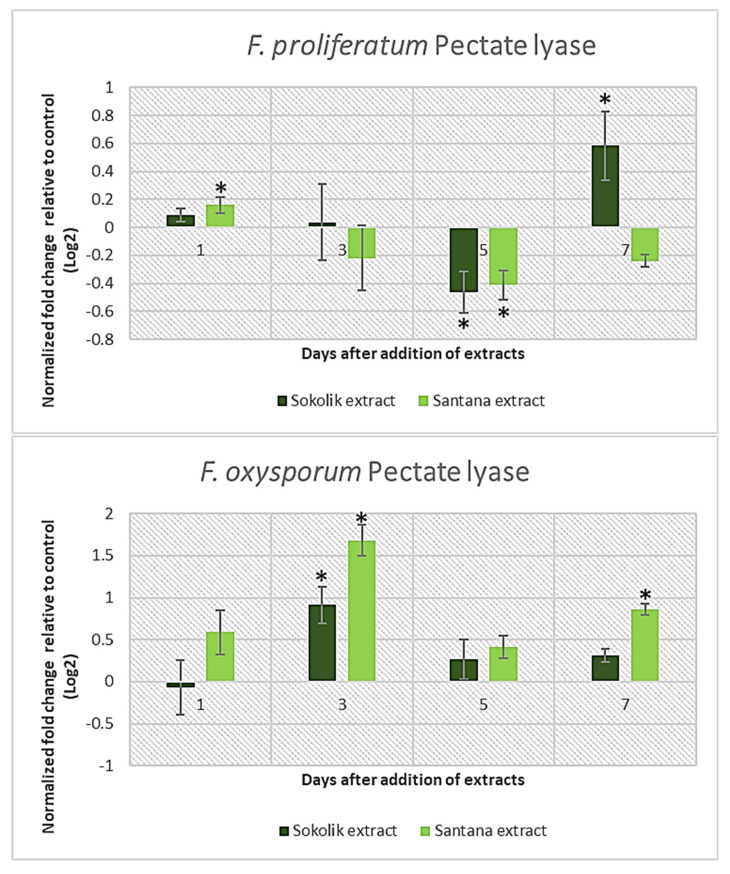
Pectate lyase gene expression of *F. proliferatum* (PEA1) and *F. oxysporum* (1757 OX) from day 1 to day 7 after the addition of the Sokolik and Santana extracts. Error bars represent the standard error. * Statistically significant (*p <* 0.05).

Methyl esterified and non-esterified pectins are mainly found in the intersections of mother–lateral roots in plants like leeks [21]. The pectin degradation is a complex process where pectin methyl-esterases and pectin lyases attack esterified pectins, whereas pectate lyases and polygalacturonases hydrolyze de-esterified pectins [22]. The expression of the pectate lyase gene in PEA1 was similar to that of the xylanase gene (Figure 3). There was a slight increase in expression in Santana extract-added cultures, which gradually reduced over time, whereas 7 days after the extract addition, Sokolik could induce an increased expression of *pl1* transcripts. All the observed three lytic genes in *F. oxysporum* 1757OX had a peculiar hike at 3 days after the extract addition.

### 2.2. Plant Infection Studies

**Figure 4:**
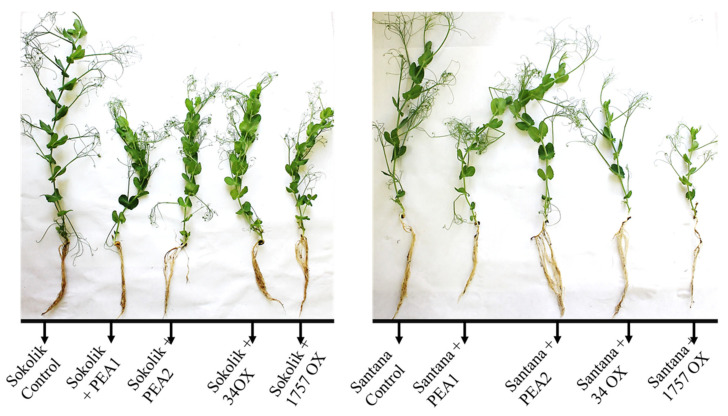
Comparison of the growth of pea plants upon infection (13th day).

A very low degree of disease symptoms was observed in the infected plants. However, all the infected plants from both cultivars showed reduced growth and a decreased number of lateral roots and leaves (Figure 4). Santana was found to have a higher growth retardation upon infection with PEA1, 34OX and 1757OX, whereas all the infected Sokolik plants had similar growth reductions. The infected and control plants cannot fully show or explain why the infected plants have reduced growths but give indirect proof of the pathogen influence on the plants, along with demonstrating how the susceptible and resistant cultivars react to the infection with the same strains.

#### 2.2.1. Cell Wall-Degrading Enzyme Assays

**Figure 5:**
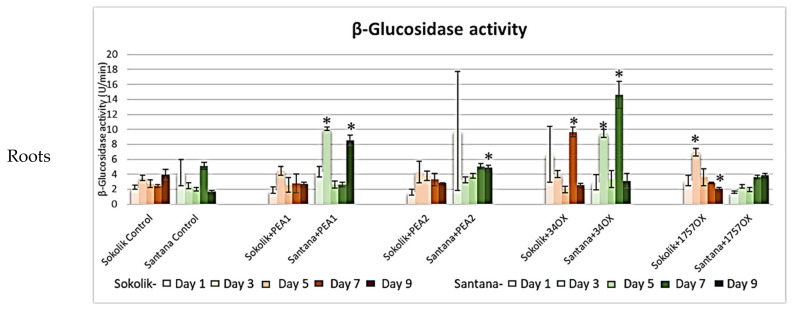

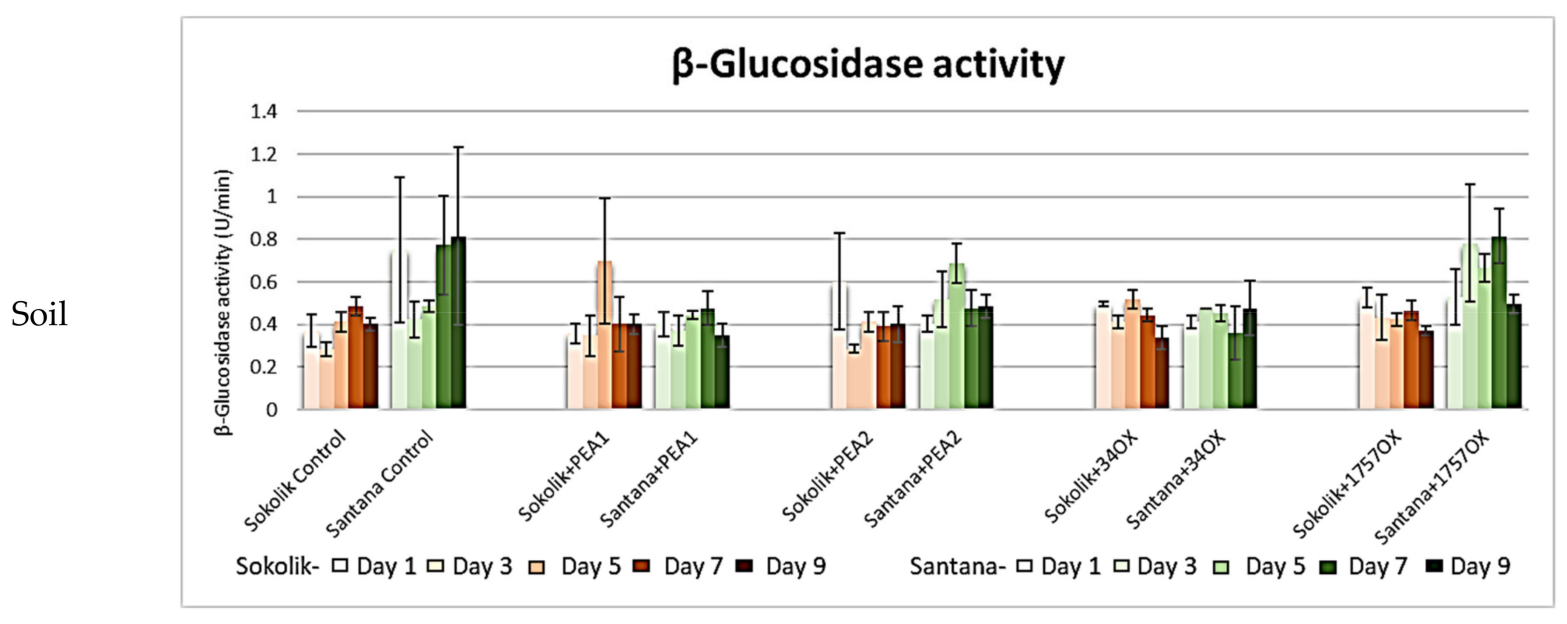
β−glucosidase activity (micrograms of P-nitrophenol produced per minute (U/min)) obtained from the roots and soil of Sokolik and Santana infected with PEA1, PEA2, 34OX and 1757OX and the control (calculated from triplicate treatments). Error bar represents the standard error. * Statistically significant (*p <* 0.05).

β-glucosidase assays showed that Santana infected with PEA1 and 34OX expressed the highest enzyme activity compared to the other two strains, especially on day 9 and day 7, respectively (Figure 5). The maximum activity of 14.8 U/min was detected in Santana upon infection with 34OX on day 7. The highest β-glucosidase activity in Sokolik was observed in 34OX-infected plants on day 7, similar to that of Santana. No significant elevation in the enzyme activity was observed in the soil samples. The plant cellulose degradation involves enzymes such as endo-β-1,4-glucanases, exo-β-1,4-glucanases and β-Glucosidases [23]. The endo-β-1,4-glucanases activity in the Sokolik roots infected with PEA1, PEA2 and 1757OX was found to be significantly higher than the controls, specifically on days 1 and 7. No statistically significant differences were observed for Santana plants, along with the results from the soil (*p* > 0.05). Likewise, no significant elevation in the activity was observed for exo-β-1,4-glucanases in both pea roots and rhizospheres, indicating that β-glucosidase is the major cell wall-degrading enzyme produced by *Fusarium* species used in this study. The graphical representations of the protease, polygalacturonase, chitinase, endo β-1,4 glucanase and exo-β-1,4-glucanase enzyme activity results are presented as Supplementary Materials (Tables S1–S5 and Figures S1–S5).

The assays for chitinase showed that the Sokolik plants infected with 1757OX were able to express much higher enzyme activity starting from day 1 to day 5, where a maximum activity of 24.8 U/min was observed on day 5. Santana infected with PEA1 and 34OX also showed increased activity compared to the control on day 3. Similar results were also observed in the soil, indicating that plants activate their defense pathways selectively based on the type of pathogen strain.

**Figure 6:**
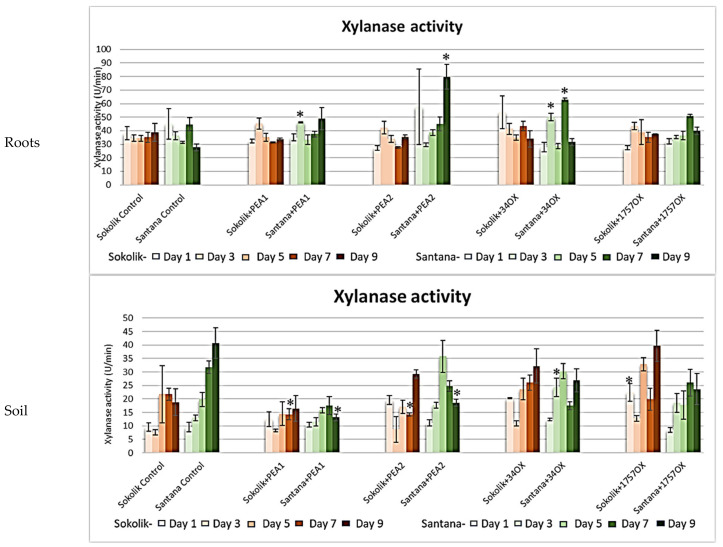
Xylanase activity (micromoles of xylose produced per minute (U/min)) obtained from the roots and soil of Sokolik and Santana infected with PEA1, PEA2, 34OX and 1757OX and the control (calculated from triplicate treatments). Error bar represents the standard error. * Statistically significant (*p <* 0.05).

All the infected Santana plants showed a higher activity of xylanase during the last days of growth (Figure 6). Although a slight increase in the activity was observed in Sokolik, especially on day 3, the values dropped on the subsequent days. There was a gradual increase in the xylanase activity in Santana in the control during the observed days. However, the soil samples with strains PEA2, 34OX and 1757OX infected in Sokolik showed a higher activity of xylanase on day 9.

**Figure 7:**
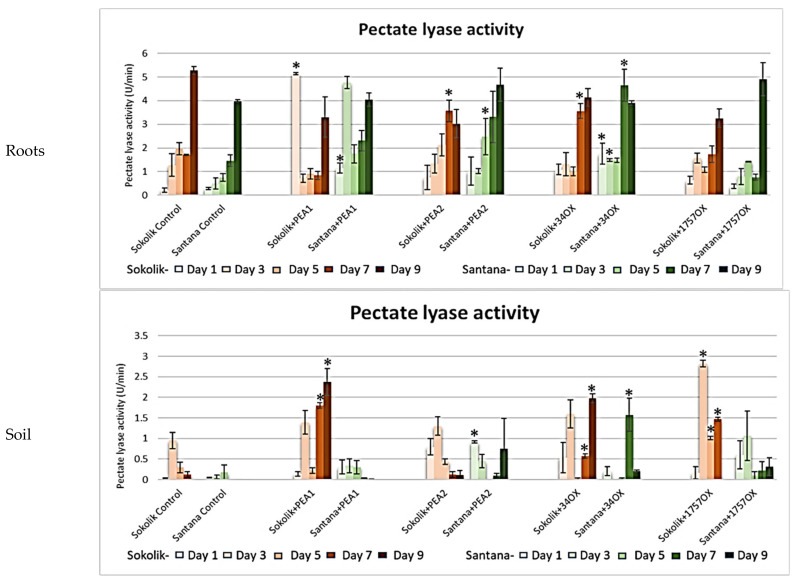
Pectate lyase activity (micromoles of galacturonic acid produced per minute (U/min)) obtained from the roots and soil of Sokolik and Santana infected with PEA1, PEA2, 34OX and 1757OX and the control (calculated from triplicate treatments). Error bar represents the standard error. * Statistically significant (*p <* 0.05).

There was a gradual increase in the pectate lyase activity in the controls of Sokolik and Santana over the observed days (Figure 7). However, the significant difference observed was on day one in Sokolik with the PEA1 infection, where a sudden increase in the activity was obtained. Comparably, it applies for Santana infected with PEA1 on day 3, signifying that PEA1 synthesizes more pectate lyase for plant cell wall pectin degradation, since the activity was not found in the control plants on the initial days. Nevertheless, the elevated activity in the soil of all the infected Sokolik plants indicated that pectate lyase is one of the most important cell wall-degrading enzymes produced by *Fusarium* species. The activity of pectate lyase was the highest in Sokolik compared to Santana, suggesting that the activity of the enzyme produced also depends on the disease resistance capacity of the host plant. A comparatively higher activity of polygalacturonase enzyme was observed in the root rhizosphere of Sokolik plants infected with all the strains except for PEA2. The activity in roots were highly variable and, hence, statistically insignificant (*p >* 0.05).

**Figure 8:**
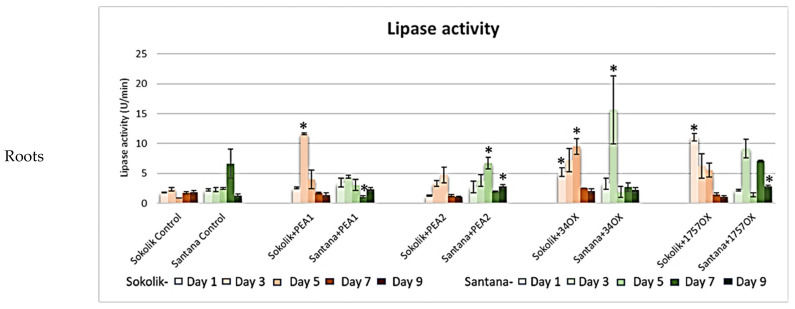

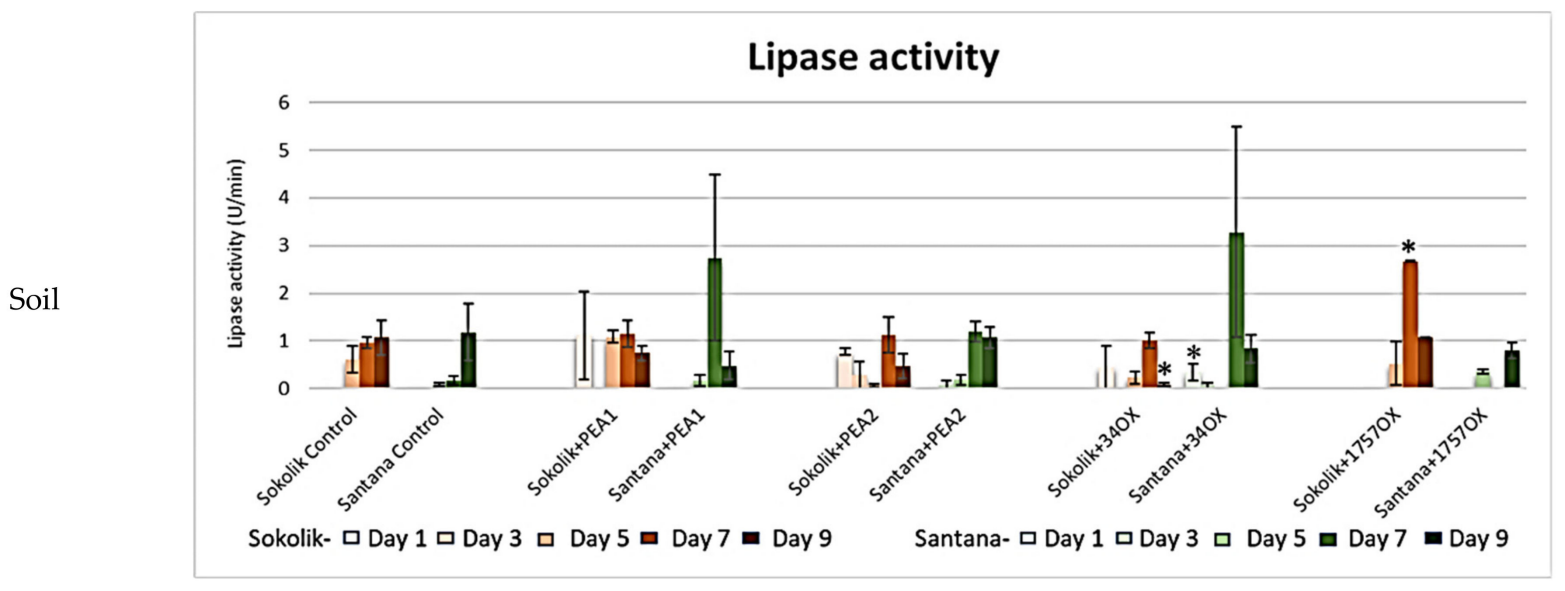
Lipase activity (micrograms of p-nitrophenol produced per minute (U/min)) obtained from the roots and soil of Sokolik and Santana infected with PEA1, PEA2, 34OX and 1757OX and the control (calculated from triplicate treatments). Error bar represents the standard error. * Statistically significant (*p <* 0.05).

The lipase activity in the Sokolik roots were found to be higher compared to the control (Figure 8). The activity was especially higher on days 1, 3 and 5 and reduced drastically on the following days. The activity was comparatively higher than the control in all the infected Santana plants, similar to that of Sokolik. A significant increase in the activity was only observed in the rhizosphere of the resistant cultivar infected with 1757OX on day 7.

The protease activity in the roots was found to be higher in both cultivars infected with the 34OX strain on day 7. Although the activities were higher compared to the control on all days, the differences were not statistically significant (*p >* 0.05). In the roots, the highest activities were observed in Sokolik plants infected with both the *F. oxysporum* strains and selectively on Santana infected with the 34OX strain during the first three observations.

**Figure 9:**
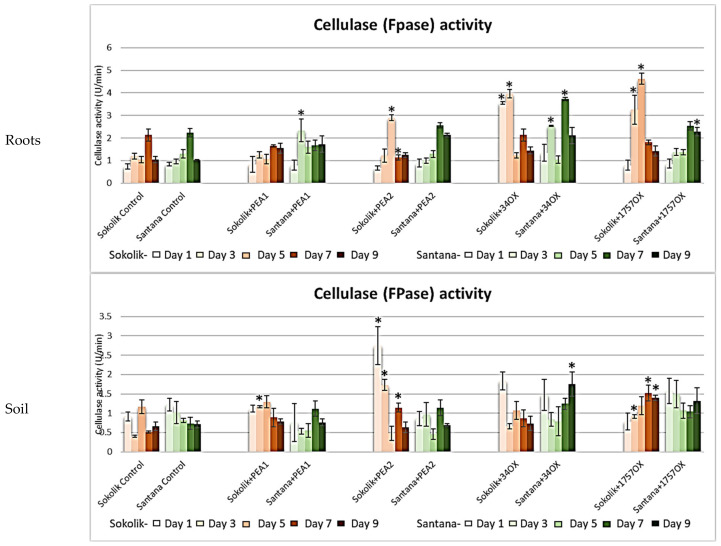
Cellulase activity obtained from the roots and soil of Sokolik and Santana infected with PEA1, PEA2, 34OX and 1757OX and the control calculated from triplicate treatments. Error bar represents the standard error. * Statistically significant (*p <* 0.05).

Exceptionally high cellulase activity was observed in Sokolik and Santana plants infected with PEA2, 34OX and 1757OX (Figure 9). The former had the highest activity during the initial stage, while the latter did on days 7 and 9. This trend has been observed for other enzymes too, where Sokolik elicited a higher increase in the enzyme activities than Santana. The highest activities of cellulase of 4U/min and 4.76 U/min were observed in Sokolik infected with *F. oxysporum*, indicating the severe pathogenicity of the strains on days 3 and 5, respectively. The cellulose activity in the soil was the highest in Sokolik+PEA2, where an activity of 5.47 U/min was reached on day 1.

The overall results from the lytic enzyme assays from the infected plants showed the preferential hydrolysis of the host plant cell wall by *Fusarium* species (Table 1). We observed different combinations of enzymes produced by the pathogen while interacting with resistant and susceptible host genotypes. Based on visible symptoms and results from the enzyme assays, it was clear that *F. oxysporum* is more pathogenic than *F. proliferatum* in peas. While interacting with resistant cultivars, the PEA1, 34OX and 1757OX strains produced an increased number of enzymes, which degrade almost all components of the cell wall, such as hemicellulose, pectins, cellulose and membrane lipids.

#### 2.2.2. Mycotoxins Quantification

**Figure 10:**
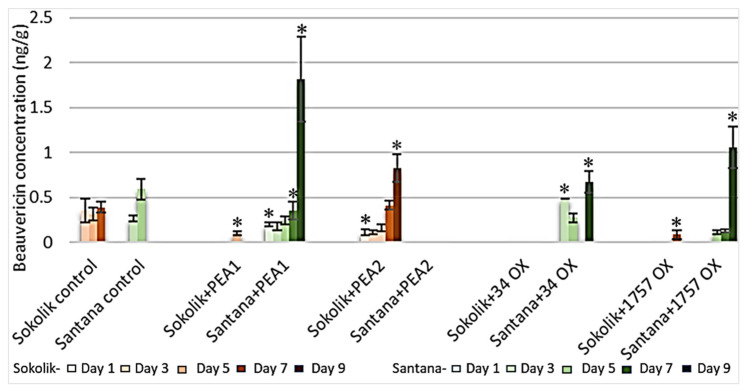
Concentration of beauvericin (ng/g) in Sokolik and Santana roots upon infection with PEA1, PEA2, 34OX and 1757OX calculated from triplicate treatments. Error bar represents the standard error. * Statistically significant (*p <* 0.05).

Only beauvericin was found in the mycotoxin analysis. Fumonisins were not detected in any of the plant samples. A substantial difference in the amount of beauvericin was observed between the two pea cultivars (Figure 10). The susceptible cultivar Santana was found to accumulate more beauvericin than the resistant cultivar. The maximum concentration of beauvericin detected in the cultivar Santana inoculated with PEA1 was 18.8 ng/g on day 9. Santana infected with PEA2 did not accumulate any toxin, the same as Sokolik, especially on the last day of observation. In contrast, no beauvericin was detected in Sokolik upon *F. oxysporum* infection. The production of beauvericin by strain PEA1 was the opposite to that observed in strain PEA2, where more beauvericin was detected in Santana plants and no/extremely low concentration was observed in Sokolik. The overall observation was that Sokolik accumulates very low amounts of beauvericin compared to the susceptible cultivar Santana.

## 3. Discussion

Cell wall-degrading enzymes are one among several mechanisms that plant–pathogenic fungi use to invade and decompose organic matters [24]. The study of different hydrolase enzymes in the plant rhizosphere indicates the soil fertility and plant productivity. The various enzyme activities in the rhizosphere can be of plant intracellular origin, released as a part of tissue degradation by various microbial communities and can be the part of microbes associated with the plant root. Although the contribution of plants in this aspect is less, it must be underlined that the current assay methods do not allow the discrimination of the enzyme’s origin [25]. Only a comparative study of enzymatic activities of plant roots and the rhizosphere could answer this complex question to an extent.

The biosynthesis of various toxins and cell wall-degrading enzymes highly depends on the culture media composition, where they can be altered by supplementing various plant cell wall components or secondary metabolites. Studies suggest that antioxidant inhibitors supplemented to the culture media could suppress the accumulation of various pathogenicity factors and lytic enzymes, making it unfavorable for *F. proliferatum* pathogenicity [26]. Similarly, it is already established that there is some kind of relationship between the amount and type of cell wall-degrading enzymes produced by pathogens and the cell wall composition of their corresponding hosts.

Although the current study does not demonstrate a direct role of any studied enzyme, it provides the basis for such research. Our previous [9] and current studies prove that the selected strains behave differently when added to the extracts of susceptible and resistant host phenotypes. The extracts from susceptible cultivars induce sudden increases in the lytic gene expression in the pathogen, whereas resistant cultivars elicit slow and reduced expressions. The monitored genes such as encoding β-glucosidase, pectate lyase and xylanase, have a reduced expression upon supplementing with the resistant pea extract, indicating that there is some kind of *Fusarium* antagonistic activity exhibited by the plant through its secondary metabolites or inhibitory proteins. Although studies suggest that the genes coding xylanase and pectate lyase enzyme are functionally redundant and the pathogen virulence or the infection machinery is independent of those genes [27,28], it is still challenging to find out how susceptible and resistant plants cope with the invasion and how their metabolites modify this ability. It has been reported that, in order to counteract and to resist the hydrolytic enzyme attack, plants deploy CWDE inhibitor proteins such as polygalacturonase-inhibiting proteins (PGIPs), pectin methylesterase inhibitor (PMEI), pectin lyase inhibitor protein (PNLIP), xylanase inhibitor protein (XIP) and xyloglucan endoglucanase inhibitor protein (XEGIP) [29]. Together with the proteins, pea plants produce several antifungal secondary metabolites, such as pisatin and lectins, which have been found to inhibit the growth of pathogens such as *Aspergillus* sp. and *Fusarium* sp. [8,30]. In planta studies have indicated that the pathogen produces an increased number of enzymes during infection in resistant cultivars and less in susceptible ones. *F. oxysporum*, being the causal agent for *Fusarium* wilt in peas worldwide, produced more enzymes compared to the *F. proliferatum* strains. The enzymes produced aid in the degradation of cellulose (cellulase and β-glucosidase), hemicellulose (xylanase), pectin (pectinase) and membrane lipids (lipase). During infection, species such as *F. phaseoli, F. graminearum, F. proliferatum* and *F. solani* generally exhibit an increased activity of β-glucosidase [31]. During infection with necrotrophic pathogens, plants also produce β-glucosidases, which have many functions, such as intermediates of cell wall lignification [32], phytohormone activation [33] and detonators of plant chemical defenses [34]. Although pectinolytic enzymes are not necessary for disease development, they were found to have some roles in the pathogenicity of the organism [35]. The pectate lyase activity was increased in both cultivars in the controls and infected plants. Especially, there was increased activity on the initial day, indicating that there is a relation between pectinolytic enzymes and *Fusarium* infection. The plant pectate lyases are associated with a diverse array of organs and cellular processes, including pollen development, flowering, leaves, stem and root expansion [36]. They are important parts of enzyme complexes controlling cell wall remodeling during various plant cellular processes. Pectate lyase methyl transferase and pectate lyase activity were reported to be higher upon treatment with auxin in Arabidopsis roots, indicating that the changes in the structure and composition of the pectin fraction are linked to the expansion of the plant cell [37]. An exceptional increase in the activity of the lipase enzyme was observed in the Sokolik plants upon infection. Lipases hydrolyze the carboxyl ester bonds in tri acyl glycerol, releasing fatty acid and glycerol [38]. Mainly, the members of the microbial community and plants produce these enzymes as a part of their metabolism. The fungal lipases degrade the fatty acids in the plasma membrane, leading to the leakage of cytoplasmic constituents and, thereby, cell death. Numerous lipases have also been identified and characterized in plants as a part of ethylene/hormone signaling involved in systemic resistance pathways, cuticle biosynthesis, the deacetylation of xylan and various secondary metabolisms [39].

The aggressiveness of *Fusarium* directly depends on the mycotoxin that they produce during host interactions [40] that function as effectors or virulence factors controlling plant pathogenesis. The difference in the levels of beauvericin in the resistant (Sokolik) and susceptible pea cultivars (Santana) indicates that the resistant genotype accumulates very low amounts of toxins compared to the susceptible one. *F. oxysporum*, being the causal agent of wilt in peas, was found to produce more beauvericin. In addition, the results suggest that the plants susceptible to *Fusarium* are also susceptible to toxin accumulation, leading to host-selective infection and disease symptoms. Similar results were obtained for *Alternaria* host-selective toxins, such as AK toxins I and II in pear cultivars, where the concentration and toxicity were higher in the susceptible cultivar compared to the resistant cultivar [41]. Beauvericin causes plasma membrane destruction through ionophoric activity, where the ion permeability of the biological membranes is altered, leading to cell death.

The pea varieties resistant to *Fusarium* wilt exhibit several defense mechanisms, where the main discriminating factor is the root endodermis [42]. Recent studies have shown that established defensive mechanisms in pea roots efficiently block the pathogen progression before vascular stele [43], and the enzymes involved in cell wall degradation vary in different pathogen races. The regions of the outer root system, including the epidermis and exodermis, an important contributor to the defense, are highly related to the disease severity and are the major determinants of resistance. Additionally, the main defense mechanisms identified were cell wall thickening of the endodermis through lignification by means of phytoalexin accumulation. This acts as a physical barrier preventing the entry of hyphae inside the roots [43]. Understanding the role of CWDEs and mycotoxins in plant–pathogen interactions is very important for the effective management of diseases, as these factors may act as host defense elicitors.

## 4. Materials and Methods

### 4.1. Fungal Gene Expression Studies

#### 4.1.1. Fungal Strains and Growth Conditions

The fungal strains were selected based on the results from the enzymatic activities carried out previously [9]. One strain each of *F. proliferatum* (PEA2) and *F. oxysporum* (1757OX) were used for the fungal gene expression studies. All the strains used for the current study were isolated from infected pea plants (*Pisum sativum* L.) during a previous study and identified based on both morphological and molecular identification techniques. Morphological parameters such as the structures of hyphae, phialides and conidia and a molecular analysis using *TEF*-1α and *ITS1-ITS2*-specific primers were used for identification [44]. The strains were selected based on the previously carried out mycotoxin production studies [9]. The PDA-grown 7-day-old cultures were added to 48 mL of the fumonisin-inducing media for the mycotoxin analysis [45]. The medium contained: malt extract 0.5 g/L, yeast extract 1 g/L, mycological peptone 1 g/L, KH_2_PO_4_ 1 g/L, MgSO_4·_7H_2_O 0.3 g/L, KCl 0.3 g/L, ZnSO_4_·7H_2_O 0.05 g/L, CuSO_4_·5H_2_O 0.01 g/L and D-fructose 20 g/L. On the 5th day of incubation, 2 mL each of the extracts from the Sokolik and Santana cultivars were supplemented to the cultures. A control was also kept without the addition of extracts. All the treatments were carried out in triplicate. The culture media were collected in 10-mL aliquots on the 6th, 8th, 10th, 12th and 14th days of incubation, and the mycelia were collected and stored at −80 °C and used later for RNA isolation.

#### 4.1.2. Plant Extract Preparation

Plant extracts were prepared according to the previously standardized protocols [46]. The leaves were collected from fully grown pea plants (Sokolik and Santana). The overnight frozen (−80 °C) samples were homogenized using a blender, and the pulp obtained was centrifuged at 12,000× *g* for 15 min. The supernatants were filtered using 0.45-μm membrane filters and stored at −20 °C until further use.

#### 4.1.3. Gene Expression Studies

The authors carried out assays for 10 enzymes in the first set of study [9]. The three genes opted for the differential expression study were selected based on their corresponding increased enzymatic activity levels obtained by the author’s previous experiments.

The total RNA was extracted and purified from around 50 mg of mycelium collected at the 6th, 8th, 10th, 12th and 14th days of incubation using the RNeasy plant mini kit (Qiagen, Hilden, Germany), followed by treatment with DNase I (RNase-free) (EURx, Gdansk, Poland). The total RNA concentration was quantified using a NanoDrop ND-1000 (Thermo Fisher Scientific, Waltham, MA, USA), and 100-ng RNA from each sample was loaded on a 1% agarose gel (50V/35 min) to check its integrity. Then, 1 μg of total RNA was reverse-transcribed into cDNA using a High-Capacity cDNA Reverse Transcription Kit (Applied Biosystems, Foster City, CA, USA). The incubation steps were as follows: 25 °C for 10 min, followed by 37 °C for 120 min and 85 °C for 5 min. All the reactions were carried out in a Bio-Rad C1000 thermal cycler. The synthesized cDNA was used as a template for RT-qPCR (SsoAdvanced Universal SYBR Green Supermix, Bio-Rad, Hercules, CA, USA), and the copy number of unambiguous transcripts for all the genes were normalized to the expression ratio by transcripts of β-tubulin. Each assay included three biological and two technical replicates of each sample, along with a negative control. The primers used in the study are given in Table 2.

The target sequences were amplified in a 10-μL reaction containing 5 μL of SsoAdvanced universal SYBR Green supermix, 500 nM of each primer and 1 μL of cDNA template (dilution 1:10). The PCR cycling conditions were as follows: initial denaturation at 95 °C for 30 s, denaturation at 95 °C 40 cycles for 15 s and annealing at 60 °C for 30 s.

### 4.2. Plant Cultivation

Seeds from both cultivars Sokolik and Santana were surface-sterilized using 15% (*v/v*) bleach in sterile distilled water for 30 s and then washed in sterile distilled water thrice to remove the bleach completely. Then, the seeds were kept on sterile wet tissue inside a Petri dish and stored in a dark chamber for three days to enhance the germination. Once the seeds were germinated, the plates were transferred to a well-lit place and kept for four more days. On day 7, the seedlings were planted on sterilized soil and were cultivated in a growth chamber at 23 °C with a 16-h photoperiod. The plants were watered as required and were fertilized with a micro–macro nutrient solution consisting of Ca(NO_3_)_2_ (4.18 g L^−1^), KNO_3_ (1.03 g L^−1^), KH_2_PO_4_ (0.35 g L^−1^), K_2_SO_4_ (0.43 g L^−1^), Mg(NO_3_)_2_ (0.51 g L^−1^), MgSO_4_ (0.63 g L^−1^) and Fe chelate (0.5 g L^−1^) once after 15 days of planting.

### 4.3. Plant Infection Studies

The plant infection studies were carried out using two strains of *F. proliferatum* (PEA1 and PEA2) and two strains of *F. oxysporum* (34OX and 1757OX). The strains were also checked for their virulence by infecting pea seedlings for the current study prior to the plant infection studies (data not shown). The spore suspensions of PEA 1, PEA 2, 34OX and 1757OX were made by flooding the corresponding single-spore isolate cultures on PDA plates with sterile distilled water. The number of conidia was counted microscopically (Nikon Optiphot-2, Tokyo, Japan) using a hemocytometer and adjusted to 1 × 10^5^ conidia mL^−1^ by dilution with sterile distilled water. From the conidia suspension, 5ml was added directly to the root rhizosphere to infect each plant that reached 30 days of growth. Each treatment consisted of three plants, and the control plants were kept separated from the infected ones. After the infection, the samples (soil and roots) were collected intermittently after 7 days.

#### 4.3.1. Enzyme Activity Assays

The soil samples collected were mixed with bicarbonate buffer (50 mM, pH 7) at a 1-g/mL concentration and were blended for 1 min [49]. The resulting slurry was centrifuged at 12,000× *g* for 15 min at 4 °C to collect the supernatant. Similarly, 1 *g* of the root samples ground with bicarbonate buffer (50 mM, pH 7) was also centrifuged at 12,000× *g* for 15 min at 4 °C. The supernatants obtained were stored at −20 °C until further use.

##### β-Glucosidase

4-nitrophenyl-β-D-glucopyranoside (PNPG) was used as a substrate for the β-glucosidase assay [50]. The reaction mixture containing 50 μL of crude enzyme and 100 μL of 2-mM PNPG was incubated for 30 min at 50 °C. Sodium carbonate of 0.1 M (100 μL) was added to the reaction mix after the incubation, and the absorbance was measured at 405 nm using a Synergy HTX Multi-Mode reader (Biotek, Vermont, VT, USA).. The reactions were carried out using biological triplicates with technical replicates. Different concentrations of 4-nitrophenol (PNP) were used to create a standard curve. The enzyme activity was expressed as the micromoles of PNP produced per minute (U/min) under the given assay conditions.

##### Chitinase

The colloidal chitin required for the assay was prepared by adding 1g of chitin powder from shrimp (Sigma Aldrich) to 12 mL of HCl and stirring overnight at 4 °C [51]. The mixture was then added to 400 mL of ice-cold 95% ethanol with continuous stirring. The resulting mixture was incubated at 25 °C overnight. Later, the mixture was centrifuged at 5000× *g* for 20 min at 4 °C, and the precipitate was collected and washed several times with sterile distilled water until the pH reached 7. The prepared colloidal chitin was refrigerated until the enzyme assay.

The chitinase activity was assayed by measuring the reducing sugars [52]. The assay was carried out by mixing 0.5 mL of both 1% colloidal chitin and the crude enzyme. After 1 h of incubation at 50 °C, 3 mL of DNS were added to the mixture to terminate the reaction. The mixture was boiled for 15 min and was centrifuged at 5000× *g* for 5 min to remove the insoluble chitin. From the supernatant, 200 μL were taken and added to the 96-well flat-bottomed plate, and the absorbance was measured at 540 nm using the Synergy HTX Multi-Mode reader. All the reactions were carried out in triplicate. A standard curve of N-acetyl-D-glucosamine was used to quantify the amount of the product released. The enzyme activity was expressed as micromoles of N-acetyl-D-glucosamine released per minute (U/min) under the given assay conditions.

##### Xylanase

Xylose was used as the reference standard for the xylanase assay. The reaction mixture containing 25 μL of 1% (*w/v*) suspension of xylan from oat spelts in 0.05-M sodium citrate buffer (pH 5.0) and 25 μL of a crude enzyme sample was incubated at 50 °C for 10 min. All the reactions were carried out in triplicate. The DNS method was used to measure the released sugars by adding 150 μL of DNS to stop the reaction and heating in a boiling water bath for 15 min [52]. Later, the absorbance at 540 nm was taken using a Synergy HTX Multi-Mode reader. The enzyme activity was expressed as micromoles of xylose released per minute (U/min) under the given assay conditions.

##### Pectate Lyase

Pectate lyase activity was measured according to our previously standardized protocol [9]. The reaction mixture contained 25 μL of the crude enzyme sample, 25 μL of 0.5% citrus pectin, 50 μL of Tris HCl Buffer (0.05 M, pH 8.0) and 50 μL of 1-M calcium chloride. The mix was incubated at 30 °C for 1 h in 96-well flat-bottomed plates. The absorbance was measured at 548 nm using the Synergy HTX Multi-Mode reader. All the reactions were carried out in triplicate. The enzyme activity was defined as the micromoles of galacturonic acid produced per min (U/min) under the given assay conditions.

##### Polygalacturonase

Polygalacturonase activity was assayed by measuring the released reducing sugars after the reaction using the dinitrosalicylic acid (DNS) method [52]. The reaction mix contained 50 μL of 0.1% polygalacturonic acid prepared in 0.05-M sodium acetate buffer (pH 5.0), 25 μL of buffer and 25 μL of culture filtrate and was incubated at 40 °C for 1 h in a water bath. All the reactions were carried out in triplicate. The absorbance was measured at 540 nm using the Synergy HTX Multi-Mode reader. A standard curve was prepared using different concentrations of galacturonic acid. One unit of enzyme activity was expressed as micromoles of galacturonic acid produced per minute (U/min) under the given assay conditions.

##### Lipase

The lipase activity was measured using P-nitro phenyl palmitate (pNPP) as the substrate and p-nitrophenol as a standard reference [53]. Twenty-five microliters of crude enzyme solution were added to 50 μL of prewarmed phosphate buffer (0.05 M, pH 8.0) containing 0.2% (*w/v*) sodium deoxycholate and 0.1% (*w/v*) gum Arabic. The resulting mixture was incubated for 10 min at 30 °C. After the incubation, the pNPP solution was added to the mix to reach a final concentration of 0.30 mM, and the mixture was again incubated at 30 °C for 3 min. All reactions were carried out in triplicate. Absorbance of the mixture was measured at a wavelength of 405 nm using the Synergy HTX Multi-Mode reader. The enzyme activity is expressed as micromoles of p-nitrophenol produced per minute under the given assay conditions.

##### Cellulase (Filter Paper Assay)

The filter paper assay was carried out using the Whatman^®^ #1 filter paper cut into ¼-inch diameter circles using a paper punch in 96-well flat-bottomed plates [54]. Of the diluted sample, 80 μL in 50-mM citrate buffer was added to the substrate and incubated at 50 °C for one hour. After the incubation, 80 μL of DNS reagent was added, and the mixture was boiled for 10 min, cooled in ice and the absorbance was measured at 546 nm. All the reactions were carried out in triplicate. One unit of FPase was defined as the micromoles of glucose released per minute (U/min).

##### Endo beta-1, 4 glucanase (CMCase)

The endo-β-1,4-glucanase activity was determined by the 3,5-dinitrosalicylic acid (DNS) method [43]. The reaction mixture containing 25 μL of crude enzyme and 0.5% (*w/v*) carboxy methyl cellulose in 75 μL of acetate buffer (0.05 M, pH 5.0) was incubated at 60 °C for 10 min. The reaction was terminated by adding 25 μL of DNS and boiling for 10 min. The mixture was cooled down to room temperature using ice. All the reactions were carried out in triplicate. Later, the absorbance was measured at 540 nm using the Synergy HTX Multi-Mode reader. One unit of endo-β-1,4-glucanase activity was defined as the micromoles of glucose released per minute (U/min) under the given assay conditions.

##### Exo-β-1,4-glucanase (avicelase)

The reaction was carried out by adding 50 μL of crude enzyme solution to 1% Avicel solution prepared in 50-mM sodium citrate buffer (pH 5.0). The resulting solution was incubated at 50 °C for 30 min. Then, 50 μL of DNS was added to the mixture, boiled for 5 min and cooled in ice for 5 min. The resulting mixture was centrifuged at 5000× *g* for 5 min to remove any unreacted substrates. The absorbance was measured at 540 nm using the Synergy HTX Multi-Mode reader. All the reactions were carried out in triplicate. The enzyme activity was expressed as micromoles of glucose released per minute (U/min) under the given assay conditions.

##### Protease

Azocasein was used as the substrate for the protease assay [55]. The dye-labeled peptides and amino acids released after the reaction with the were measured after the assay. From the sample, 25 μL was added to the equal volume of 0.1% azocasein prepared in 0.2-M Tris-HCL buffer (pH 7.4). The solution was incubated at 75 °C for 1 h, and the reaction was terminated by adding 100 μL of 5% trichloroacetic acid (TCA) to the enzyme–substrate mixture. The mixture was centrifuged at 2000× *g* for 10 min at room temperature to remove the coagulated protein. The supernatant obtained was then added to an equal volume of 0.5-N NaOH solution, and the absorbance was measured at 440 nm using the Synergy HTX Multi-Mode reader. The blank was obtained by mixing the TCA to the substrate prior to the enzyme addition. All the reactions were carried out in triplicates. The activity of the enzyme was expressed as the absorbance at 440 nm.

### 4.4. Mycotoxins Analysis

Certified analytical standards of mycotoxins (fumonisins B_1-3_ and beauvericin), other reagents, water and organic solvents of high purify for the chromatographic analysis were purchased from Sigma-Aldrich (Steinheim, Germany). A small amount (0.20 g) of each plant root was extracted using 1.0 mL of methanol:water (3:1, *v/v*) by shaking on an orbital shaker (24 h) and by sonication for 20 min. After centrifugation (Eppendorf, Hamburg, Germany) at 15,000× *g* for 10 min, the extracts were filtered through a 0.22-μm membrane (Chromafil PET 20/15/MS, Macherey-Nagel, Germany) and transferred to chromatography vials. The mycotoxin concentration was analyzed for each variant of the plant material using an UPLC™ system (Acquity, Waters, Milford, MA, USA) connected with a photodiode array detector (PDA) and a triple-quadrupole mass spectrometer (TQD; Waters Micromass, Manchester, UK) according to the methods described in detail earlier [56,57]. The samples were collected from biological triplicates, and qualitative and quantitative analyses of the mycotoxins were performed in three analytical replicates.

### 4.5. Statistical Analysis

The statistical analyses were made with the Origin Pro 2020 program (OriginLab Corporation, Massachusetts, MA, USA). The data obtained from lytic enzyme production from the plant infection experiments were analyzed with a one-way ANOVA with three replicates. Dunnett’s test was used to determine the significant differences between the treatments and the control, with *p <* 0.05.

## Figures and Tables

**Table 1:** Major enzymes produced by *F, proliferatum* (PEA1 and PEA2) and *F. oxysporum* (34OX and 1757OX) upon interaction with resistant (Sokolik) and susceptible (Santana) pea cultivars.

Cell Wall-Degrading Enzymes	* F. proliferatum * PEA1		* F. proliferatum* PEA2		*F. oxysporum* 34OX		* F. oxysporum* 1757OX	
	Sokolik	Santana	Sokolik	Santana	Sokolik	Santana	Sokolik	Santana
Xylanase				√	√	√	√	
Protease					√	√		
Lipase	√			√	√	√	√	√
Polygalacturonase	√				√		√	
Pectin Lyase	√				√		√	
Chitinase							√	
Endo β-1, 4 glucanase	√		√				√	
Exo-β-1,4-glucanase								
β-glucosidase		√			√	√	√	
Cellulase		√	√		√	√	√	√

**Table 2:** List of primers used in the study.

	PEA1	1757 OX
β-Glucosidase (*bgl1* gene)	BG1f-CTATCCCTCGCTGCAAGAAC BG1r-GTGGGCAACAAGAAGGTTGT	BGf-CCCAAGCAACTCCGAGGTTT BGr-TGCTGGAGCCGACGAAAATG
Pectate lyase (*pl1* gene)	PL1f-CTAGCCTCTGTTTGCCAAGG PL1r-TCAGCATGAGAAACGGTGAG	PL4f-GGTGAGCAAGTTTCTCTCGACT PL4r-CACTGGTCTGCTTGAGGGTG
Xylanase (*xyl4* (*F. proliferatum*) and *xyl2* gene (*F. oxysporum*))	XYL4f-CCATCAACTATGGCGGTTCT XYL4fr-GTAGACGGTGCCCTTGTGTT	XYL2f-CAGGTCGTCAACTTTGCTCA XYL2r-TTAACCCACTGAGGGAGCTG
β-tubulin	Fpbtf-ACATCCAGACAGCCCTTTGTG Fpbtr-AGTTTCCGATGAAGGTCGAAGA [47]	Fobtf-TTCTGCTGTCATGTCCGGTGT Fobtr-TCAGAGGAGCAAAGCCAACCA [48]

## Data Availability

The data presented in this study are available in the article and Supplementary Materials.

## Supplementary Materials

The following are available online at https://www.mdpi.com/article/10.3390/ijms22189888/s1: Tables S1–S5: Mean and standard error (SE) of protease (S1), polygalacturonase (S2), chitinase (S3), endo *β*-1, 4 glucanase (S4), exo *β*-1, 4 glucanase (S5) and activity assays obtained from the soil and roots of Sokolik and Santana infected with PEA1, PEA2, 34 OX and 1757 OX and the control. Figures S1–S5: Graphical representations of protease (S1), polygalacturonase (S2), chitinase (S3), endo *β*-1, 4 glucanase (S4) and exo *β*-1, 4 glucanase (S5) activities obtained from the roots and soil of Sokolik and Santana infected with PEA1, PEA2, 34 OX and 1757 OX and the control.
