# Supplementary material for: Dynamics of Fusarium Mycotoxins and Lytic Enzymes during Pea Plants’ Infection
Source: Int J Mol Sci. 2021 Sep 13;22(18):9888. doi: 10.3390/ijms22189888 (PMC8467997; doi:10.3390/ijms22189888)
Supplement: Supplementary file 1 [file ijms-22-09888-s001.zip › ijms-1311964-supplementary.pdf]

Table S1. Mean and standard error (SE) of protease activity obtained from soil and roots of Sokolik and Santana infected with PEA1, PEA2, 34 OX, 1757 OX and control. *Statistically significant (p<0.05).

		Day 1		Day 3		day 5		Day 7		Day 9	
		Soil	Roots	Soil	Roots	Soil	Roots	Soil	Roots	Soil	Roots
Sokolik Control	(U/min)	0.06	0.06	0.06	0.06	0.06	0.06	0.06	0.06	0.07	0.06
	SE	0.00	0.00	0.00	0.00	0.00	0.00	0.00	0.00	0.00	0.00
Santana Control	(U/min)	0.07	0.06	0.09	0.06	0.07	0.06	0.10	0.08	0.08	0.06
	SE	0.00	0.01	0.00	0.00	0.00	0.00	0.01	0.00	0.01	0.00
Sokolik+PEA1	(U/min)	0.06	0.07	0.06	0.07	0.06	0.06	0.06	0.06	0.06	0.06
	SE	0.00	0.00	0.00	0.00	0.00	0.00	0.00	0.00	0.00	0.00
Santana+PEA1	(U/min)	0.07	0.07	0.07*	0.07	0.07	0.07	0.06*	0.06*	0.07	0.07*
	SE	0.00	0.00	0.00	0.02	0.00	0.00	0.00	0.00	0.00	0.00
Sokolik+PEA2	(U/min)	0.06	0.06	0.09	0.06	0.07	0.06	0.06	0.06	0.08	0.06
	SE	0.00	0.00	0.03	0.00	0.00	0.00	0.00	0.00	0.01	0.00
Santana+PEA2	(U/min)	0.07	0.06	0.08*	0.06	0.07	0.07	0.07*	0.06*	0.07	0.06
	SE	0.00	0.01	0.00	0.00	0.00	0.00	0.00	0.00	0.00	0.00
Sokolik+34OX	(U/min)	0.07*	0.07	0.08	0.07	0.08*	0.07	0.06	0.11*	0.07	0.06
	SE	0.00	0.01	0.00	0.01	0.00	0.01	0.00	0.00	0.00	0.00
Santana+34OX	(U/min)	0.09	0.06	0.09	0.07	0.09*	0.06	0.07*	0.11*	0.08	0.07*
	SE	0.01	0.00	0.01	0.02	0.01	0.00	0.00	0.00	0.00	0.00
Sokolik+1757OX	(U/min)	0.08*	0.06	0.10*	0.08	0.10*	0.07	0.06	0.06	0.08	0.06
	SE	0.00	0.01	0.01	0.01	0.01	0.01	0.00	0.00	0.00	0.00
Santana+1757OX	(U/min)	0.06*	0.06	0.08	0.06	0.07	0.06	0.07	0.07	0.08	0.07
	SE	0.00	0.00	0.00	0.00	0.00	0.01	0.00	0.00	0.00	0.00

Table S2. Mean and standard error (SE) of polygalactuonase activity obtained from soil and roots of Sokolik and Santana infected with PEA1, PEA2, 34 OX, 1757 OX and control. *Statistically significant (p<0.05).

		Day 1		Day 3		day 5		Day 7		Day 9	
		Soil	Roots	Soil	Roots	Soil	Roots	Soil	Roots	Soil	Roots
Sokolik Control	(U/min)	1.12	0.44	0.75	1.36	0.70	0.75	0.99	1.24	1.12	1.36
	SE	0.58	0.21	0.27	0.65	0.37	0.40	0.28	0.12	0.27	0.65
Santana Control	(U/min)	0.63	1.85	0.32	1.67	0.38	0.32	0.69	0.81	0.81	1.54
	SE	0.28	0.06	0.22	0.06	0.12	0.06	0.37	0.18	0.11	0.32
Sokolik+PEA1	(U/min)	1.93	0.20	0.93	0.20	2.22*	0.07	1.48	1.24	1.24	0.99
	SE	1.40	0.12	0.40	0.12	0.27	0.06	0.43	0.16	0.06	0.18
Santana+PEA1	(U/min)	0.44	0.20*	0.69	1.05	1.05	0.38	1.97	0.87	1.36	1.12
	SE	0.11	0.06	0.34	0.27	0.27	0.06	0.27	0.32	0.93	0.06
Sokolik+PEA2	(U/min)	1.55	0.38	0.63	0.26	0.56	1.12	0.75	1.30	1.36	0.81
	SE	0.56	0.06	0.21	0.00	0.40	0.58	0.12	0.31	0.37	0.11
Santana+PEA2	(U/min)	0.50	0.93	0.44	0.81*	0.44	0.81*	1.48	1.30	1.12	1.18
	SE	0.22	0.50	0.11	0.18	0.21	0.18	0.62	0.27	0.32	0.11
Sokolik+34OX	(U/min)	2.77	0.63	0.07	0.63	0.56	0.99	1.42	1.24	1.42	2.01
	SE	0.25	0.18	0.28	0.32	0.22	0.49	0.32	0.16	0.16	0.94
Santana+34OX	(U/min)	0.38	0.26*	0.44	1.67	0.26	0.87*	1.05	1.54	1.97	1.05
	SE	0.12	0.11	0.37	0.12	0.11	0.06	0.12	0.11	0.64	0.16
Sokolik+1757OX	(U/min)	0.38	0.44	0.44	2.16	0.39	2.22	1.79	1.81	2.10*	0.93
	SE	0.12	0.21	0.11	0.16	0.06	0.37	0.43	0.93	0.28	0.25
Santana+1757OX	(U/min)	0.56	0.01*	0.99	0.75*	0.63	0.32	1.05	1.05	1.30	1.85
	SE	0.27	0.00	0.32	0.06	0.21	0.06	0.25	0.32	0.44	0.64

Table S3. Mean and standard error (SE) of chitinase activity obtained from soil and roots of Sokolik and Santana infected with PEA1, PEA2, 34 OX, 1757 OX and control. *Statistically significant ($p<0.05$).

		Day 1		Day 3		day 5		Day 7		Day 9	
		Soil	Roots	Soil	Roots	Soil	Roots	Soil	Roots	Soil	Roots
Sokolik Control	(U/min)	3.91	9.23	5.56	9.91	4.09	9.03	6.29	9.39	4.00	8.68
	SE	0.39	0.80	1.19	0.66	0.39	0.38	1.82	0.49	0.41	1.11
Santana Control	(U/min)	4.09	10.54	5.44	11.28	5.39	10.84	5.23	10.00	5.69	6.76
	SE	0.39	0.81	0.34	2.11	1.26	0.41	0.83	8.36	0.26	0.31
Sokolik+PEA1	(U/min)	4.71	8.32	3.91	10.32	5.48	9.10	4.18	11.76	6.04	8.53
	SE	0.24	0.44	0.09	0.71	0.74	0.47	0.24	1.56	1.25	0.69
Santana+PEA1	(U/min)	5.37	9.32	4.09	15.73*	4.09	9.67	4.85	9.32	4.43	8.82*
	SE	1.44	0.38	0.49	0.31	0.18	0.43	0.51	0.43	0.57	0.40
Sokolik+PEA2	(U/min)	4.78	7.96	4.27	10.06	3.91	11.89	3.91	7.89	4.25	6.97
	SE	0.92	0.47	0.53	0.89	0.39	0.62	0.24	0.85	0.65	0.36
Santana+PEA2	(U/min)	5.46	9.19	5.19	10.45	3.56	10.72	4.53	10.53	3.56	9.66*
	SE	0.21	1.41	0.50	0.12	0.18	0.48	0.41	0.47	0.09	0.59
Sokolik+34OX	(U/min)	5.16	11.80	3.56	11.07	4.62	10.16	4.53	9.66	3.56	7.47
	SE	0.64	2.05	0.71	1.28	0.24	0.87	0.00	1.26	0.36	0.12
Santana+34OX	(U/min)	5.56	9.62	8.39*	16.75*	6.15	10.81	4.00	12.89	5.62	7.54
	SE	0.67	0.77	0.62	0.41	1.65	1.56	0.31	0.87	0.68	0.51
Sokolik+1757OX	(U/min)	5.26	10.17	6.72	15.23*	5.19	24.68*	4.53	9.24	4.52	7.25
	SE	1.01	0.07	1.55	0.90	0.39	7.42	0.15	0.61	0.60	1.05
Santana+1757OX	(U/min)	4.87	8.53	5.48	10.19	5.65	9.75	6.40	16.33	5.85	9.88*
	SE	0.87	0.54	0.63	0.65	1.13	1.00	0.54	2.38	0.94	0.31

Table S4. Mean and standard error (SE) of endo β- 1,4- glucanase activity obtained from soil and roots of Sokolik and Santana infected with PEA1, PEA2, 34 OX, 1757 OX and control. *Statistically significant ($p<0.05$).

		Day 1		Day 3		day 5		Day 7		Day 9	
		Soil	Roots	Soil	Roots	Soil	Roots	Soil	Roots	Soil	Roots
Sokolik Control	(U/min)	6.96	2.54	6.65	8.20	5.09	5.91	2.04	4.11	1.98	6.94
	SE	1.01	1.33	0.66	1.18	0.58	0.95	0.38	0.87	1.04	0.66
Santana Control	(U/min)	7.90	7.32	6.15	5.45	4.99	3.07	6.35	5.97	3.89	5.25
	SE	0.78	0.24	0.52	1.21	0.42	1.63	0.71	0.37	0.07	0.31
Sokolik+PEA1	(U/min)	5.06	5.88*	6.31	6.02	5.58	6.71	3.72	8.12*	5.22	7.42
	SE	1.28	0.11	1.24	1.40	1.21	0.45	0.52	1.18	0.04	1.36
Santana+PEA1	(U/min)	4.74	1.73	2.32	8.29	5.92	6.15	3.66	6.25	4.76	8.18
	SE	0.56	1.73	1.29	0.84	1.24	0.26	0.76	0.20	0.40	1.26
Sokolik+PEA2	(U/min)	6.67	5.11	6.62	7.59	8.70	6.83	4.15	7.78*	4.11	7.38
	SE	1.15	0.80	0.73	1.02	1.13	0.92	0.68	1.25	0.82	0.15
Santana+PEA2	(U/min)	4.51	5.37	3.18	6.42	5.75	5.15	0.00*	4.57	0.14	5.59
	SE	0.38	0.80	1.59	0.42	0.27	2.60	0.00	1.68	0.14	2.20
Sokolik+34OX	(U/min)	6.10	4.96	5.53	7.87	5.83	7.05	5.85*	5.56	1.73	4.87
	SE	0.68	0.54	0.10	0.99	0.40	0.94	0.85	0.86	0.89	1.33
Santana+34OX	(U/min)	6.77	4.90	7.28	8.90	6.60	6.96	4.99	6.66	4.95	2.65
	SE	0.74	0.97	1.23	0.84	0.36	1.03	0.37	0.92	0.69	0.51
Sokolik+1757OX	(U/min)	7.21	6.04*	6.34	8.44	6.00	6.55	3.33	9.53*	5.05	5.71
	SE	0.48	0.29	0.31	0.37	0.58	0.81	0.56	0.30	1.00	0.73
Santana+1757OX	(U/min)	7.33	6.76	5.20	6.63	6.82	2.62	7.40	5.97	5.08	7.02
	SE	0.69	0.30	0.58	1.09	0.71	1.59	0.90	0.52	0.69	0.86

Table S5. Mean and standard error (SE) of exo β- 1,4- glucanase (avicelase) activity obtained from soil and roots of Sokolik and Santana infected with PEA1, PEA2, 34 OX, 1757 OX and control. *Statistically significant ($p<0.05$).

		Day 1		Day 3		day 5		Day 7		Day 9	
		Soil	Roots	Soil	Roots	Soil	Roots	Soil	Roots	Soil	Roots
Sokolik Control	(U/min)	31.87	22.59	25.85	38.85	26.60	34.18	37.80	33.95	37.24	38.60
	SE	0.50	1.99	3.70	1.40	2.51	3.74	1.00	5.17	3.56	1.91
Santana Control	(U/min)	35.82	39.77	22.09	42.76	28.36	35.52	22.84	45.87	35.17	34.38
	SE	3.20	4.20	5.26	2.46	3.08	2.09	4.81	2.13	2.50	2.23
Sokolik+PEA1	(U/min)	3.88*	36.79*	22.34	39.07	16.03	39.73	34.82	31.12	38.08	33.55
	SE	2.49	2.23	1.76	0.35	2.65	0.35	3.86	3.94	1.58	4.58
Santana+PEA1	(U/min)	35.39	34.52	31.12	38.94	32.88	33.63	22.84	34.40	40.53	37.40
	SE	1.57	2.31	2.23	3.87	0.50	2.23	1.65	4.05	0.74	0.50
Sokolik+PEA2	(U/min)	26.76	26.10	26.60	36.14	25.85	37.21	32.63	34.89	35.57	33.63
	SE	9.42	1.33	2.01	1.15	2.01	2.21	5.02	1.25	2.82	2.14
Santana+PEA2	(U/min)	33.76	36.17	21.08	32.63*	34.57	38.46	38.58*	45.24	34.38	33.51
	SE	4.47	2.44	6.40	1.33	8.63	1.63	1.33	5.69	1.65	2.68
Sokolik+34OX	(U/min)	29.36	32.63*	24.35	29.36	25.76	33.53	26.35*	35.18	31.37	36.07
	SE	0.00	2.89	3.80	5.34	7.32	4.45	1.30	2.94	0.50	3.77
Santana+34OX	(U/min)	29.62	36.31	21.33	43.07	43.38*	34.96	40.67*	42.27	30.38	39.13
	SE	2.89	2.15	0.25	0.87	2.51	3.77	1.14	0.13	4.41	1.96
Sokolik+1757OX	(U/min)	13.94	35.14*	22.09	45.38	29.87	41.08	38.16	32.88	30.67	26.03
	SE	4.48	1.00	2.62	1.30	3.90	7.62	0.52	2.19	5.14	8.26
San-tana+1757OX	(U/min)	31.62	25.60*	33.03	37.95	21.58	40.06	28.36	40.80	21.58*	38.21
	SE	3.86	1.51	3.29	1.67	0.90	3.17	3.90	1.06	2.79	1.92

Roots

Soil

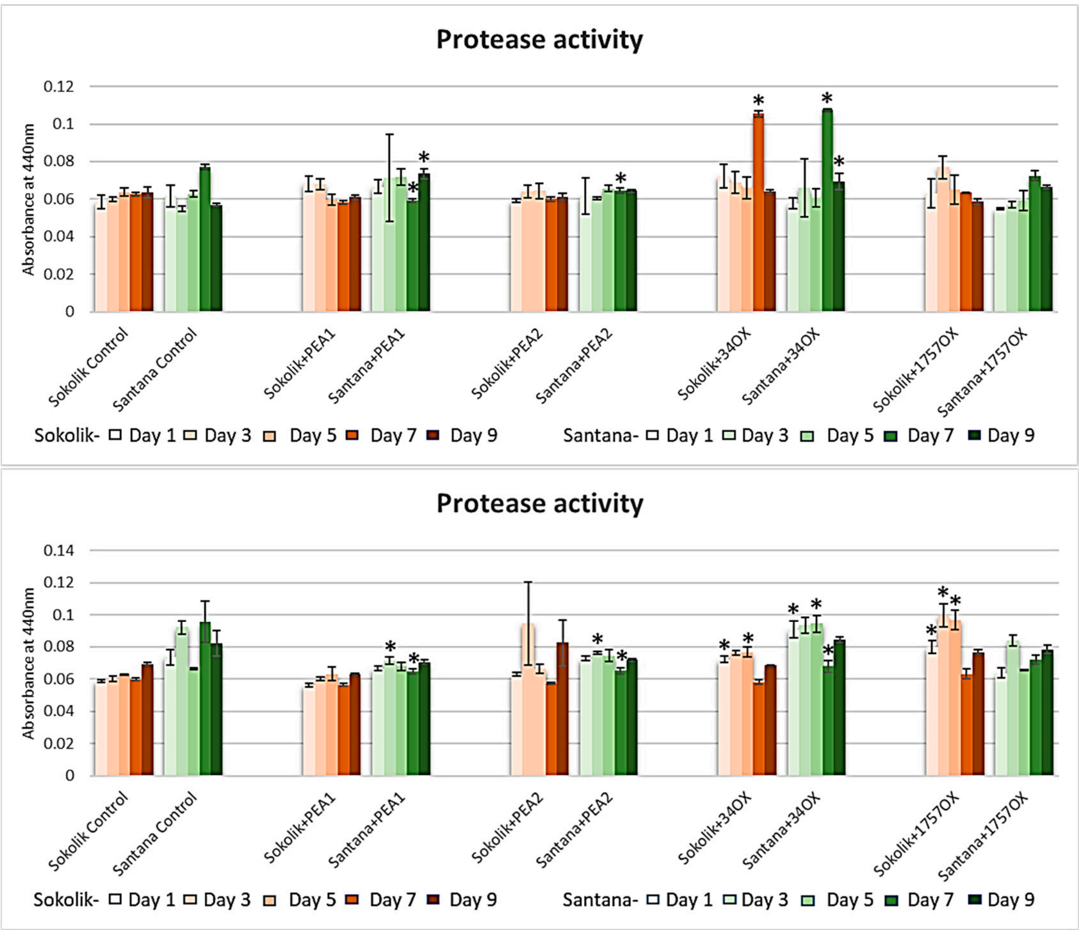

Figure S1. Protease activity obtained from roots and soil of Sokolik and Santana infected with PEA1, PEA2, 34 OX, 1757 OX and control. Error bar represents standard error. *Statistically significant ($p<0.05$).

Roots

Soil

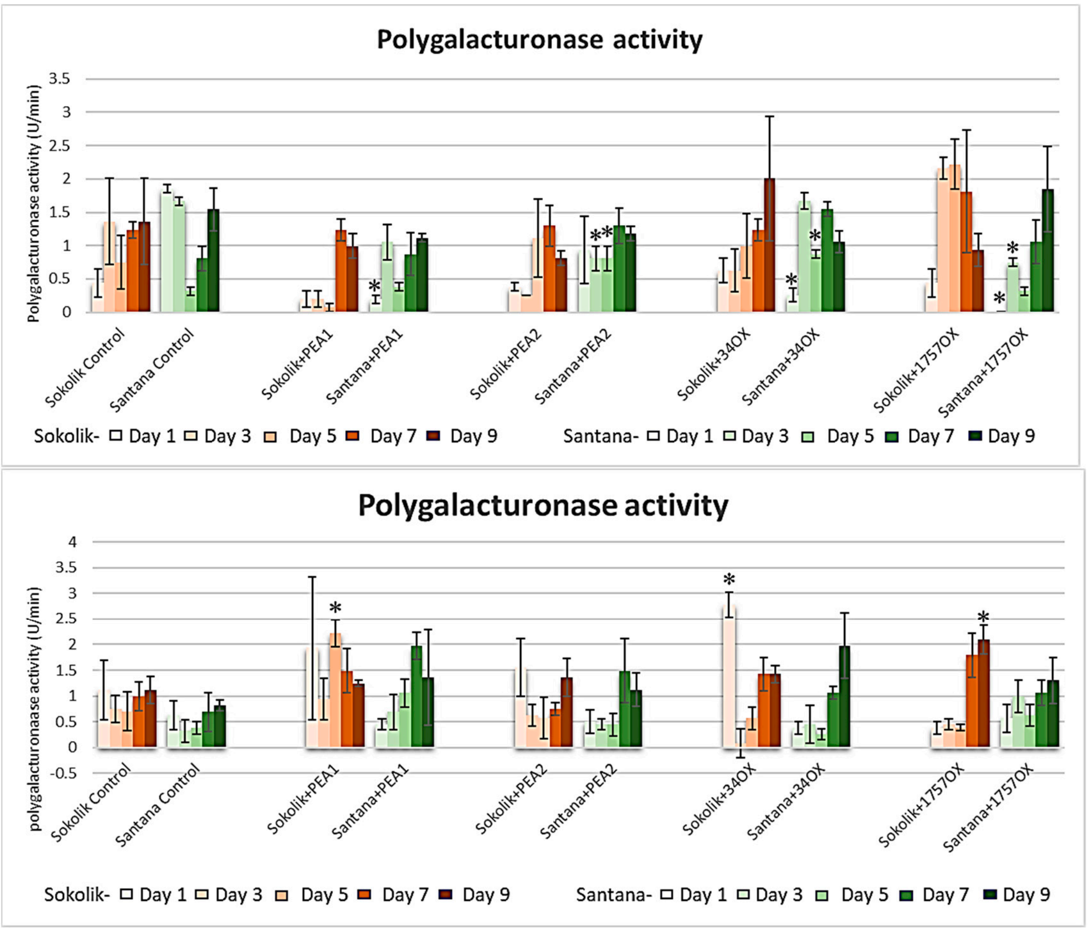

Figure S2. Polygalacturonase activity obtained from roots and soil of Sokolik and Santana infected with PEA1, PEA2, 34 OX, 1757 OX and control. Error bar represents standard error. *Statistically significant ($p<0.05$).

Roots

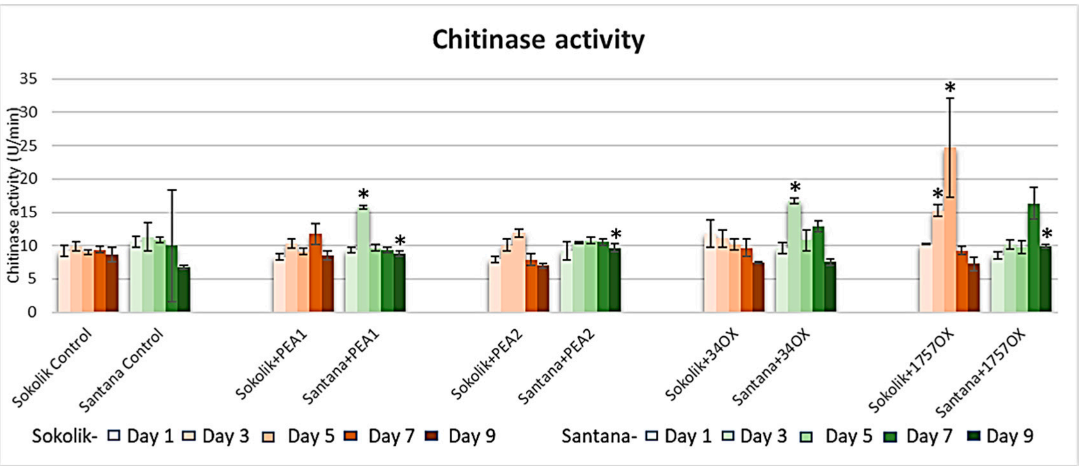

Soil

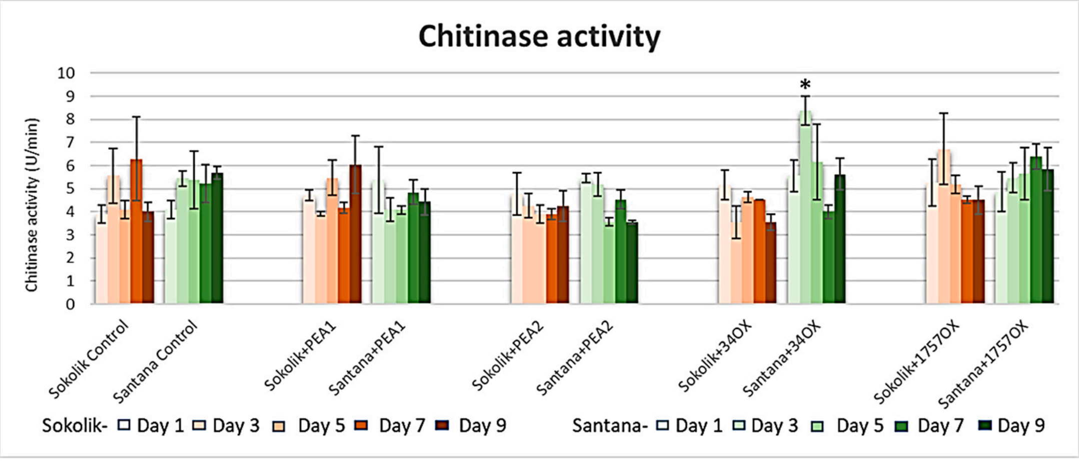

Figure S3. Chitinase activity obtained from roots and soil of Sokolik and Santana infected with PEA1, PEA2, 34 OX, 1757 OX and control. Error bar represents standard error. *Statistically significant ($p<0.05$).

Roots

Soil

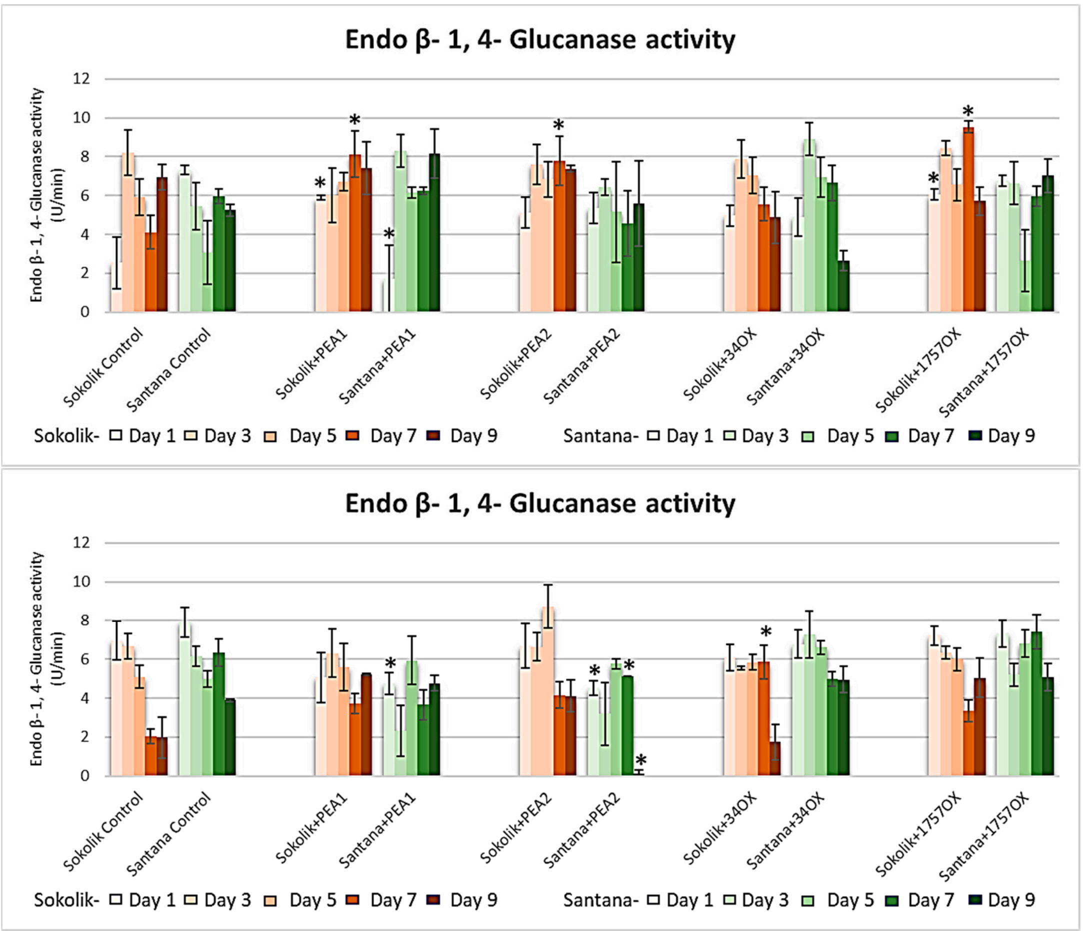

Figure S4. Endo β-1, 4-glucanase activity obtained from roots and soil of Sokolik and Santana infected with PEA1, PEA2, 34 OX, 1757 OX and control. Error bar represents standard error. *Statistically significant ($p<0.05$).

Roots

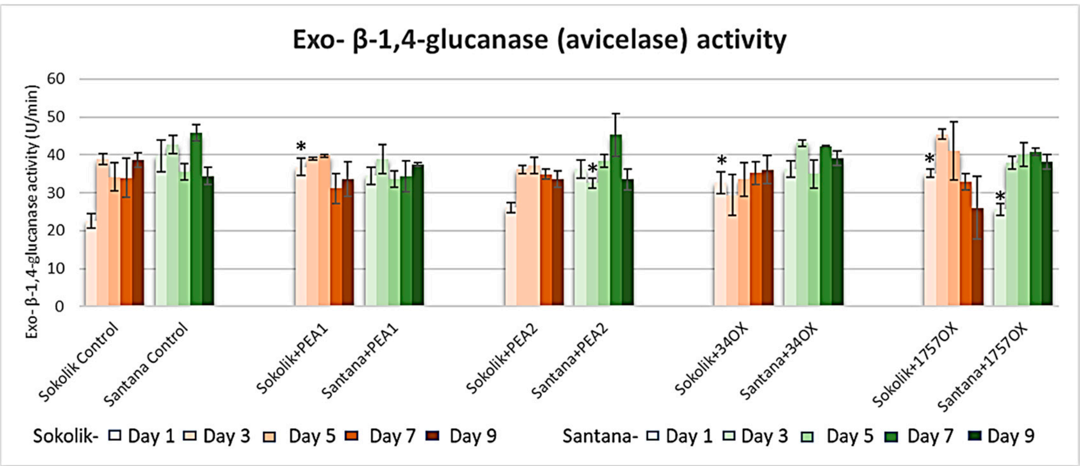

Soil

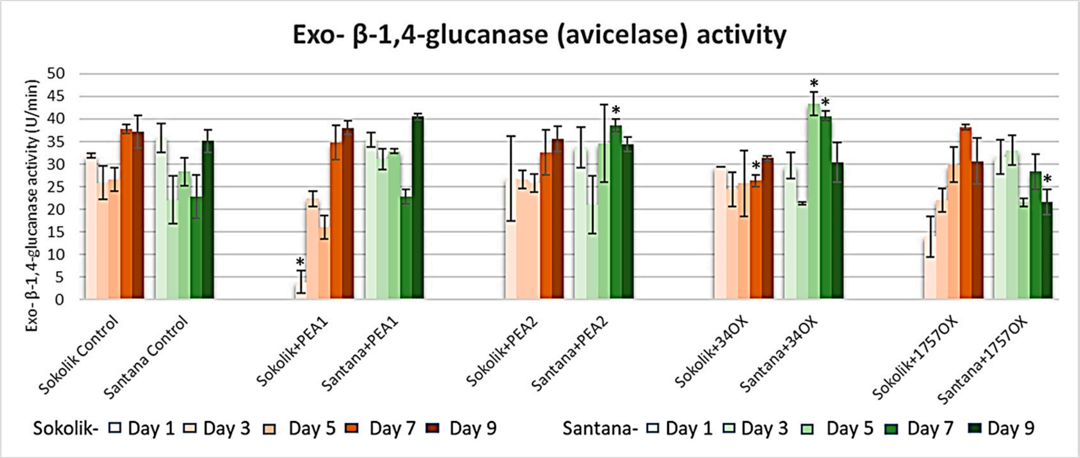

Figure S5. Exo β-1, 4-glucanase (Avicelase) activity obtained from roots and soil of Sokolik and Santana infected with PEA1, PEA2, 34 OX, 1757 OX and control. Error bar represents standard error. *Statistically significant ($p<0.05$).
